# Industrial Energy-Related CO_2_ Emissions and Their Driving Factors in the Yangtze River Economic Zone (China): An Extended LMDI Analysis from 2008 to 2016

**DOI:** 10.3390/ijerph17165880

**Published:** 2020-08-13

**Authors:** Linlin Ye, Xiaodong Wu, Dandan Huang

**Affiliations:** 1 School of Geography Science, Nantong University, Nantong 226000, China; 17826155927@163.com; 2 Cryosphere Research Station on the Qinghai-Tibet Plateau, State Key Laboratory of Cryospheric Sciences, Northwest Institute of the Eco-Environment and Resources, Chinese Academy of Sciences, Lanzhou 730000, China; wuxd@lzb.ac.cn

**Keywords:** Yangtze River Economic Zone, carbon emissions, LMDI, investment intensity, R&D intensity, R&D efficiency

## Abstract

As the world’s largest developing country in the world, China consumes a large amount of fossil fuels and this leads to a significant increase in industrial energy-related CO_2_ emissions (IECEs). The Yangtze River Economic Zone (YREZ), accounting for 21.4% of the total area of China, generates more than 40% of the total national gross domestic product and is an important component of the IECEs from China. However, little is known about the changes in the IECEs and their influencing factors in this area during the past decade. In this study, IECEs were calculated and their influencing factors were delineated based on an extended logarithmic mean Divisia index (LMDI) model by introducing technological factors in the YREZ during 2008–2016. The following conclusions could be drawn from the results. (1) Jiangsu and Hubei were the leading and the second largest IECEs emitters, respectively. The contribution of the cumulative increment of IECEs was the strongest in Jiangsu, followed by Anhui, Jiangxi and Hunan. (2) On the whole, both the energy intensity and R&D efficiency play a dominant role in suppressing IECEs; the economic output and investment intensity exert the most prominent effect on promoting IECEs, while there were great differences among the major driving factors in sub-regions. Energy structure, industrial structure and R&D intensity play less important roles in the IECEs, especially in the central and western regions. (3) The year of 2012 was an important turning point when nearly half of these provinces showed a change in the increment of IECEs from positive to negative values, which was jointly caused by weakening economic activity and reinforced inhibitory of energy intensity and R&D intensity.

## 1. Introduction

With the development of the world economy, a large amount of energy has been consumed, and conspicuous CO_2_ has been released to the air, further accelerating global warming rates. The Intergovernmental Panel on Climate Change (IPCC) has predicted that the global temperature will increase by 1.4–5.8 °C during 1990–2100 [1]. Climate warming can lead to increasing heatwaves and extreme events (e.g., storms and floods) as well as heat-related morbidity, mortality and diseases [2]. Obviously, CO_2_ emissions from the combustion of fossil fuels, as the main contributor to greenhouse gases, can affect public health. Fossil fuel energy consumption and CO_2_ emissions have been regarded as a threat to human health [3,4].

China is the largest developing country, with the largest population in the world. According to the International Energy Agency (IEA), China’s CO_2_ emissions made up one-third of the global CO_2_ emissions from fossil fuel combustion in 2017. To achieve sustainable development, the Chinese government announced that China would reduce their CO_2_ emission intensity (carbon emission per unit of gross domestic product (GDP)) by 40 to 45% in 2020 compared with the that of 2005 [5].

Disentangling the driving factors of CO_2_ emissions is helpful to implement a national carbon emission reduction target. Structural decomposition analysis (SDA) and index decomposition analysis (IDA) are two international common factor decomposition techniques in energy-related environmental analysis. Compared with SDA, IDA requires less data and is appropriate for time-series analysis [6]. The logarithmic mean Divisia index (LMDI) based on IDA can disentangle multiple factors, process incomplete datasets and eliminate the residual error [7,8]. Therefore, it is the most widely used decomposition method to investigate the drivers of CO_2_ emission.

In China, CO_2_ emissions have attracted many research interests. There are several studies to explore the influencing factors of CO_2_ emissions in China [6,9,10,11]. Xu et al. [12] broke down the influencing factors of carbon emissions at a multi-regional scale (29 provinces and municipalities); the results suggested that the inhibitory effect of energy structure was the strongest in the eastern region, followed by the central region and the western region. Zhang et al. [10] performed a decomposition analysis of the drivers of CO_2_ emissions during 2000–2016, and the results showed that the effect of the industrial structure changed from promotion to inhibition in the period since 2010; the effect of economic activity increased and then decreased as a result of changes in the GDP growth rate. These findings suggest that the factors contributing to CO_2_ emissions differ among regions and time periods in China. However, most of these studies only considered several conventional factors on CO_2_ emissions, including carbon emission coefficient, energy structure, energy intensity, industrial structure, economic output, population and urbanization [6,13], while technological factors, namely R&D efficiency and R&D intensity, were largely ignored. Recently, the LMDI model has been extended by introducing the three factors to explain changes in carbon emissions [14,15,16,17,18,19] and it was suggested that investment intensity displays a promotional effect, whereas R&D efficiency and R&D intensity can bring either positive or negative effects on carbon emissions.

**Figure 1:**
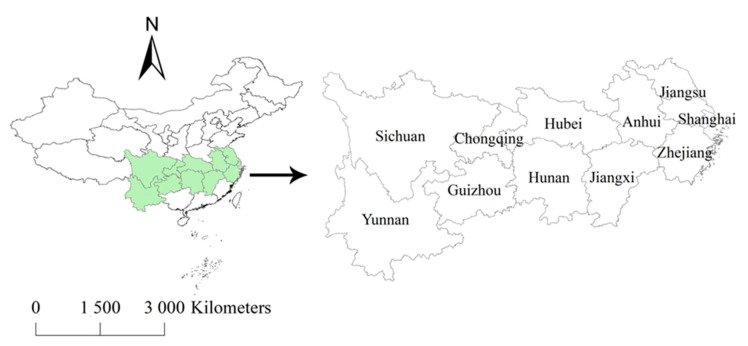
Location of the Yangtze River Economic Zone (YREZ) in China.

The Yangtze River Economic Zone (YREZ) (Figure 1) accounts for 21.4% of the total area of China. The YREZ is the home of around 40% of the national population, and this region generates more than 40% of the total national GDP. The YREZ is composed of nine provinces and two municipalities (Table A1). Shanghai, Jiangsu and Zhejiang provinces are located in the developed east coastal region, representing the forefront of transformation and upgrading of industrial structure and being confronted with energy shortage, over-population and other environmental problems. Chongqing, Sichuan, Guizhou and Yunnan provinces are located in the western region, with great endowment of natural resources, such as natural gas, shale gas and geothermal energy. Anhui, Jiangxi, Hubei and Hunan provinces are situated in the central region, which is considered as the production base of energy, agriculture and raw materials in China. During the process of industrial transfer, the central region is responsible for the linkage between the eastern and western regions. These sub-regions in the YREZ have great differences in socio-economic conditions, natural resource endowments and energy consumption. To promote reasonable energy management and environmental protection, the collaborative and dislocation development of YREZ has become a national strategy. It is evident that the YREZ region is becoming increasingly important. That is, the fastest-growing economy in YREZ is likely to increase CO_2_ emissions in the near future, consequently accelerating climate change. YREZ has a low and flat topography, a developed water network and high precipitation [20]. Therefore, the frequency and severity of water-related disasters may increase due to climate change [20] and then affect public health. However, as a major source of CO_2_ emissions, the industrial energy-related CO_2_ emissions (IECEs) in YREZ during recent years are unclear, and the driving factors of IECE changes in sub-regions within YREZ remain unknown. These knowledge gaps hinder the cooperative development of the YREZ region and also are barriers to the national carbon emission mitigation target. This study aims to calculate IECEs from 2008 to 2016 using an extended LMDI model and to explore the different factors contributing to IECEs in the YREZ. The main goals of the study are to address the following questions: (1) What are the spatial and temporal patterns of IECEs within the YREZ region during 2008–2016? (2) Which factor promotes and counteracts the increase in the IECEs? The results will clarify the IECE patterns and their driving factors, which will provide a scientific basis for carbon emission control strategies in the future.

## 2. Methodology and Data Source

### 2.1. Calculation Method of IECEs

The purpose of this study was to calculate industrial energy-related CO_2_ emissions (IECEs). According to the classification of the economic sector in the China Statistical Yearbooks, the primary, secondary and tertiary industry were calculated. The primary industry is constituted by “Agriculture, forestry, animal husbandry, fishery and water conservancy”; the secondary industry is constituted by “industry” and “construction”; the tertiary industry is constituted by “transport, storage and post”, “wholesale and retail trades and hotels and catering services” and “other sectors” [21]. According to the China Energy Statistics Yearbooks, eight categories of major energy were considered, including raw coal, coke, crude oil, gasoline, kerosene, diesel, fuel oil and natural gas. IECEs were calculated according to the Intergovernmental Panel on Climate Change guidelines [22]:

(1) $$C=\sum_{i=1}^{3}\sum_{j=1}^{8}C_{ij}=\sum_{i=1}^{3}\sum_{j=1}^{8}AE_{ij}\cdot LCV_{j}\cdot C_{Qj}\cdot Q_{j}\cdot \frac{44}{12}$$

where *C* is the total IECE (unit: 10^4^ tons); *C_ij_* is the IECE by energy *j* in industry sector *i* (unit: 10^4^ tons); *AE_ij_* is the consumption of energy *j* in industry sector *i* (unit: 10^4^ tons, 10^8^ m^3^); *LCV_j_* is the average low caloric value of the energy *j* (unit: MJ/t or MJ/m^3^); *CQ_j_* is the calorific value of carbon in units representing energy *j* (tC/TJ); *O_j_* is the carbon oxidation rate in the process of fossil fuel combustion (unit: %).

### 2.2. LMDI Decomposition Method

An extended LMDI model [19] was used to break down the IECE changes into the following nine factors:

(2) $$\begin{array}{l} C=\sum_{i}\sum_{j}\frac{C_{ij}}{E_{ij}}\cdot \frac{E_{ij}}{E_{i}}\cdot \frac{E_{i}}{GDP_{i}}\cdot \frac{GDP_{i}}{GDP}\cdot \frac{GDP}{R}\cdot \frac{R}{F}\cdot \frac{F}{GDP}\cdot \frac{GDP}{P}\cdot P\\ =\sum_{i}\sum_{j}CF_{ij}\cdot ES_{ij}\cdot EI_{i}\cdot IS_{i}\cdot RE\cdot RI\cdot II\cdot EO\cdot P \end{array}$$

Definitions of variables in Equation (2) are presented in Table 1.

Then, the changes in carbon emission from the year 0 to the year T were broken down into nine effects:

(3) $$\Delta C_{tot}=\sum_{i}\sum_{j}\left(\Delta C_{CF}+\Delta C_{ES}+\Delta C_{EI}+\Delta C_{IS}+\Delta C_{RE}+\Delta C_{RI}+\Delta C_{II}+\Delta C_{EO}+\Delta C_{P}\right)$$

(4) $$\Delta C_{CF}=w(C_{ij}{}^{T},C_{ij}{}^{0})\cdot In\left(\frac{CF_{ij}{}^{T}}{CF_{ij}{}^{0}}\right)$$

(5) $$\Delta C_{ES}=w(C_{ij}{}^{T},C_{ij}{}^{0})\cdot In\left(\frac{ES_{i}{}^{T}}{ES_{i}{}^{0}}\right)$$

(6) $$\Delta C_{EI}=w(C_{ij}{}^{T},C_{ij}{}^{0})\cdot In\left(\frac{EI_{i}{}^{T}}{EI_{i}{}^{0}}\right)$$

(7) $$\Delta C_{IS}=w(C_{ij}{}^{T},C_{ij}{}^{0})\cdot In\left(\frac{IS_{i}{}^{T}}{IS_{i}{}^{0}}\right)$$

(8) $$\Delta C_{RE}=w(C_{ij}{}^{T},C_{ij}{}^{0})\cdot In\left(\frac{RE^{T}}{RE^{0}}\right)$$

(9) $$\Delta C_{RI}=w(C_{ij}{}^{T},C_{ij}{}^{0})\cdot In\left(\frac{RI^{T}}{RI^{0}}\right)$$

(10) $$\Delta C_{II}=w(C_{ij}{}^{T},C_{ij}{}^{0})\cdot In\left(\frac{II^{T}}{II^{0}}\right)$$

(11) $$\Delta C_{EO}=w(C_{ij}{}^{T},C_{ij}{}^{0})\cdot In\left(\frac{EO^{T}}{EO^{0}}\right)$$

(12) $$\Delta C_{P}=w(C_{ij}{}^{T},C_{ij}{}^{0})\cdot In\left(\frac{P^{T}}{P^{0}}\right)$$

(13) $$w(C_{ij}{}^{T},C_{ij}{}^{0})=\left\{ \begin{array}{l} \frac{\left(C_{ij}{}^{T}-C_{ij}{}^{0}\right)}{InC_{ij}{}^{{}_{T}}-InC_{ij}{}^{{}_{{}^{0}}}},C_{ij}{}^{T}\neq C_{ij}{}^{0}\\ C_{ij}{}^{T},C_{ij}{}^{T}=C_{ij}{}^{0}\neq 0 \end{array}\right.$$

Δ*C_tot_* and Δ*C_CF_* are the total effects of carbon emission decomposition and carbon emission coefficient; the carbon emission coefficients of the same energy are assumed to be unchanged during the study period. Only the results of the remaining eight factors are shown: Δ*C_ES_* and Δ*C_EI_* are the energy structure effect and energy intensity effect; Δ*C_IS_* and Δ*C_RE_* are the industrial structure effect and R&D efficiency effect; Δ*C_RI_* and Δ*C_II_* are the R&D intensity effect and investment intensity effect; Δ*C_EO_* and Δ*C_P_* are the economic output effect and population effect.

### 2.3. Data Sources

The study is based on annual data relating to the period 2008–2016. The data for the three industrial added values—R&D expenditure, total fixed-asset investment, and population—were collected from China Statistical Information (http://www.stats.gov.cn/). The data for energy consumption and CO_2_ emission in sub-regions in YREZ were derived from the China Energy Statistical Yearbook. To eliminate the influence of price changes, we deflated the raw data at current prices to the base year (2008) using GDP growth index (the preceding year = 100).

## 3. Results and Discussion

### 3.1. Economy in the YREZ

**Figure 2:**
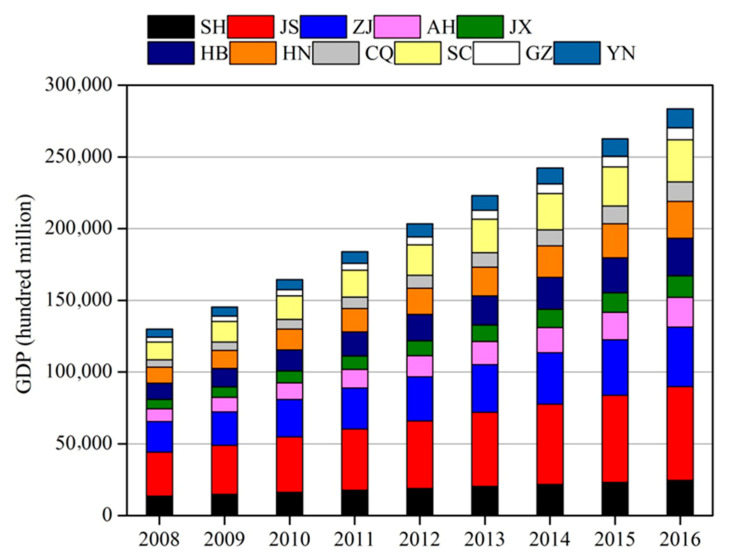
GDP in the YREZ during 2008–2016.

The economy increased in the YREZ from 2008 to 2016, with an average annual growth rate of 10.25%. Compared with the developed eastern region, the central and western regions had a weaker economic base, with a higher average annual growth rate (10.93%–13.22%) (Figure 2). From 2012, China has been undergoing a deep socio-economic transformation; the growth rate of the GDP was first observed to decrease since the reform and reopening in China [23]. This economic development phenomenon was termed the Chinese new normal. The average annual growth rate of GDP in the YREZ decreased from 11.84% during 2008–2012 to 8.67% during 2012–2016.

### 3.2. Changes in IECEs

**Figure 3:**
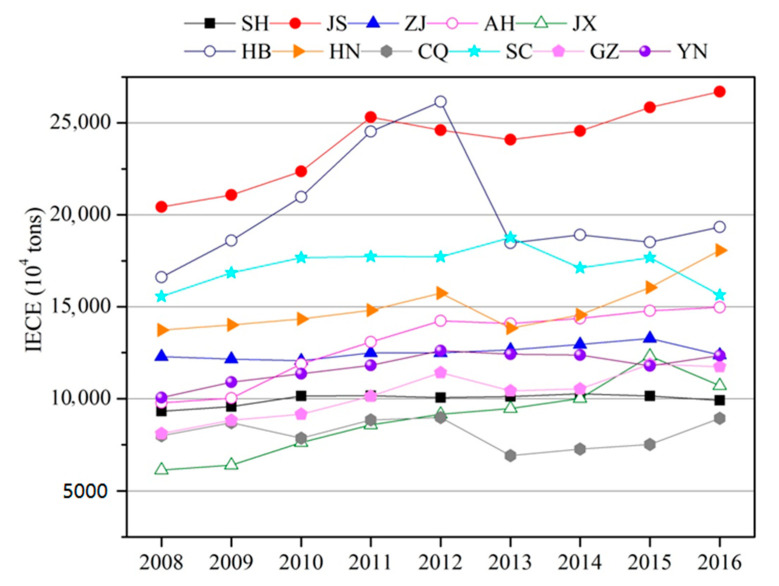
Spatial and temporal changes in IECEs in the YREZ during 2008–2016.

The energy-related carbon emission calculation method proposed by the IPCC is the most preferred method to calculate CO_2_ emissions, and it provides a scientific basis for macro-decisions regarding economy development. To validate the computed data, a comparison was made between our results and the data from multi-resolution emission inventory for China [24], which suggested that the results are similar. The total carbon emissions in the YREZ were 160,728.26 × 10^4^ tons in 2016 (Figure 3). It was reported that the national industry-related carbon emission was 6844 million tons in the same year [25], indicating that the contribution of the YREZ’s carbon emission in China was 23%. Therefore, the YREZ plays an important role in the national IECEs. Among the sub-regions, JS was the biggest IECE emitter, accounting for 15.82% of the YREZ’s total IECEs, followed by HB (13.40%) and SC (11.43%). JX and CQ were the smallest IECE emitters in the YREZ, accounting for 5.87% and 5.40%, respectively.

The total carbon emissions in the YREZ increased from 129,981.41 × 10^4^ tons in 2008 to 160,728.26 × 10^4^ tons in 2016, with an annual average growth rate of 2.79%. Due to the slow-down in China’s economic growth, CO_2_ emissions have remained relatively stable since 2012 [26]. The annual average increasing rate of IECEs in the YREZ was 5.86% during 2008–2012, while it decreased to −0.28% during 2012–2016.

### 3.3. Driving Factors of IECEs

Based on the LMDI model, the IECEs in YREZ during 2008–2016 were broken down, and the annual and cumulative effect of the driving factors, i.e., carbon emission factors, energy structure, energy intensity, industrial structure, GDP per capita, population, R&D efficiency, R&D intensity and investment intensity, were calculated (Table 2). The cumulative increment of IECEs in the YREZ was 30,746.85 × 10^4^ tons during 2008–2016 (JS > AH > JX > HN > GZ > HB > YN > CQ > SH > ZJ > SC). The cumulative increment of IECEs in sub-regions showed a decreasing trend from 2008–2012 to 2012–2016, with the exception of HN. Overall, the cumulative increment of IECEs decreased from 33,171.94 × 10^4^ tons during 2008–2012 to −2425.09 ×10^4^ tons during 2012–2016 in the YREZ.

**Figure A1:**
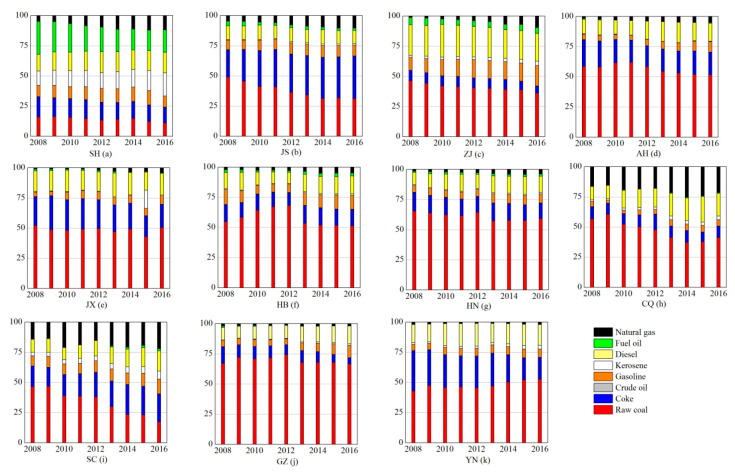
Proportion of energy structure in the YREZ during 2008–2016. (**a**) SH; (**b**) JS; (**c**) ZJ; (**d**) AH; (**e**) JX; (**f**) HB; (**g**) HN; (**h**) CQ; (**i**) SC; (**j**) GZ; (**k**) YN.

The energy structure reflects the influence of changes in the proportions of energy category on carbon emissions [12]. The energy structure changes (Figure A1) resulted in a reduction in IECEs of −2726.95 × 10^4^ tons in the YREZ during 2008–2016. The effect of energy structure on IECEs in all these provinces were negative, with the exception of JS. It is generally acknowledged that the CO_2_ emission factor of raw coal and coke was the highest, followed by crude oil and then natural gas. JS is a large industrial province that relies heavily on fossil fuels. The annual average growth rate of coke was the fastest (5.65%) in the YREZ. Therefore, energy structure had a positive effect on IECEs in JS. The inhibitory effect of energy structure was the strongest in SC, which is rich in clean energy. The annual average decreasing rate of raw coal in SC was the fastest in the YREZ (−11.02%), mitigating IECEs by−738.09 × 10^4^ tons during the study period. However, the absolute values of these numbers are relatively small, suggesting that the effect of the energy structure is weak.

Energy intensity is defined as the ratio of energy consumption and GDP, which reflects the economic efficiency of the energy utilization. The improved energy efficiency can make full use of energy and thus plays an important role in curbing carbon emissions. The energy intensity led to a reduction in IECEs of −97,438.69 × 10^4^ tons in the YREZ during 2008–2016, suggesting that the energy intensity has a strong inhibitory effect. The effect may be driven by emission reduction policies issued by the government. It is worth noting that HN, CQ and SC, where the inhibition effect of energy intensity during 2012–2016 was weaker than in 2008–2012, decreased by 54%, 27% and 17%. The trend may be explained by the following reason: compared with the developed eastern region, HN, CQ and SC encountered technical bottlenecks in improving energy efficiency.

**Figure A2:**
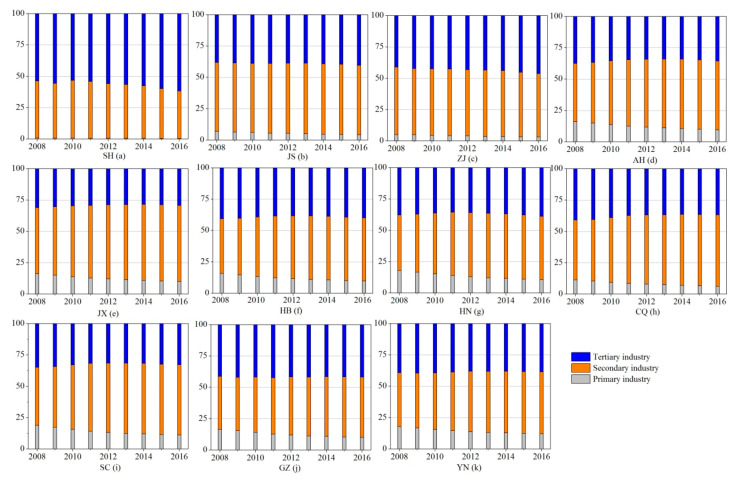
Industry structure in the YREZ during 2008–2016. (**a**) SH; (**b**) JS; (**c**) ZJ; (**d**) AH; (**e**) JX; (**f**) HB; (**g**) HN; (**h**) CQ; (**i**) SC; (**j**) GZ; (**k**) YN.

The industrial structure effect on IECEs was 9192.42 × 10^4^ tons in the YREZ. The effect was negative in SH and ZJ, whereas it was positive in the other provinces from 2008 to 2016. The proportion of industry structure (Figure A2) showed that the primary, secondary and tertiary industry accounted for 8.5%, 51.8% and 39.7% in the YREZ. SH and ZJ, in the post-industrial stage, had a relatively lower proportion of secondary industry and higher proportion of tertiary industry. Compared with the eastern region in the post-industrial stage, the central and western region are in the process of industrialization. Therefore, the industrial structure was dominated by the secondary industry. The secondary industry emits a large amount of CO_2_. For example, in SC, the proportion of secondary industry increased from 46.30% in 2008 to 56.14% in 2016, with the fastest average annual growth rate of 2.47%, showing the strongest promotional effect of the industrial structure.

R&D efficiency reflects whether there was a production expansion effect of R&D expenditure. When R&D investment is used to create new manufacturing techniques, it will lead to an increase in output and energy consumption. When R&D investment is used for reducing fossil fuel energy-related CO_2_ emissions, IECEs can be mitigated. In the present study, R&D efficiency decreased IECEs by 114,185.58 × 10^4^ tons in the YREZ, indicating that there was a decreasing return to scale in R&D expenditure. The change in IECEs related to the R&D efficiency effect during 2012–2016 was significantly lower than the same effect during 2008–2012.

R&D intensity is defined as the proportion of R&D expenditure in fixed asset investment. Due to data availability, this paper calculated R&D intensity as a whole, instead of dividing R&D into general R&D and green R&D [10]. The R&D intensity effect on IECEs was 30,078.68 × 10^4^ tons in the YREZ, indicating that the effect was positive. However, in GZ, the effect was negative, with a value of −6055.21 × 10^4^ tons. The effect of R&D intensity varied considerably with time period. In more than half of these provinces, the effect changed from promotion to inhibition from 2008–2012 to 2012–2016. It was reported that R&D intensity presented an obvious mitigating effect in SH [14], HB [19] and on the national level [15]. Taken together, the effect of R&D intensity varied considerably, which may be associated with changes in the performers of R&D activities [17] and technical barriers.

The effect of investment intensity on IECEs is ambiguous. If more money was invested into factories with heavy energy consumption, it would lead to an increase in IECEs. On the contrary, if money was invested in technology-intensive industries and industries that use clean energy, the increase in investment intensity would curb IECEs [15]. The investment intensity led to an increase in IECEs of 84,627.72 × 10^4^ in the YREZ. It generally has an important promotional effect on IECE increases, with the exception of SH. The effect of investment intensity on IECEs was strongest (16,759.30 × 10^4^ tons) in HB, while the contribution was −2474.14 × 10^4^ tons in SH. The tertiary industry accounted for more than 50% in SH; the proportion of investment flowing into industries with high technology may be higher. Therefore, energy efficiency in the production process may be improved and therefore partly abate IECEs. These results are in agreement with a previous finding [18], i.e., the co-effect of R&D expenditure and fixed asset investment is oriented towards an “new round of economic growth” rather than an “energy–saving effect”.

GDP per capita is defined as economic output. Generally, the relationship between economic output and environmental pollution conforms to the classical environmental Kuznets curve (EKC) hypothesis. In other words, before the turning point, carbon emissions and economic output are positively correlated. The economic output led to an increase in IECEs of 114,185.58 × 10^4^ tons in YREZ, particularly in JS, HB, SC, HN and AH, reaching 17,168.49 × 10^4^, 16,688.20 × 10^4^, 14,485.32 × 10^4^, 11,233.26 × 10^4^ and 10,630.61 × 10^4^ tons, respectively. In 2012, China’s economy entered into the new normal mode, characterized by a change from high-quantity development to high-quality development. Consequently, the economic output effect on IECEs during 2012–2016 was weaker in comparison with that during 2008–2012.

Population drove growth in energy demand for construction and transport infrastructure; hence, the increase in population will stimulate growth in carbon emissions, and a positive effect of population on carbon emission is expected. GZ is a traditional large province which exports labor services to the developed regions and the average annual growth rate of the population is negative. On the contrary, SH and ZJ are the most developed regions in China, which can provide more employment opportunities. Thus, there is a high population growth rate. IECEs increased significantly due to population growth in SH and ZJ (2450.98 × 10^4^ tons and 1079.54 × 10^4^ tons, respectively) during 2008–2016. This result is consistent with a previous study, suggesting that population has a greater impact in economically developed provinces [27].

CO_2_ emissions from rural areas have not been addressed in the present study. It has been reported that biomass and coal account for around 40% and 19% of the total residential energy use, respectively [28,29]. Furthermore, black carbon (BC) caused by low efficiency of combustion of fossil fuels and bio-fuels has multiple adverse impacts on public health [30,31]. Since China has a large population in rural areas, estimation of energy use and CO_2_ emission in rural China are important in the future work.

## 4. Conclusions

In the present study, the IECEs were calculated in the YREZ during 2008–2016; an extended LMDI method was then used to break down the increase in IECEs into nine factors, namely carbon emission coefficient, energy structure, energy intensity, industrial structure, R&D efficiency, R&D intensity, investment intensity, GDP per capita and population scale. The main conclusions are as follows:

(1) IECEs increased significantly in the YREZ from 2008 to 2016, with an annual average growth rate of 2.79%. The cumulative increase in IECEs was 30,746.85 × 10^4^ tons. Economic output was the main factor that increased IECEs, with a contribution of 114,185.58 × 10^4^ tons. The promotional effects of population were weaker, with a contribution of 7534.49 × 10^4^ tons. R&D efficiency and energy intensity were the main factors that decreased IECEs, with contribution of −114,706.40 × 10^4^ tons and −97,438.69 × 10^4^ tons, respectively. The inhibitory effect of energy structure was only −2726.95 × 10^4^ tons.

(2) The emission and cumulative increment of IECEs in sub-regions in the YREZ was divergent. JS was the largest carbon emitter, followed by HB and SC. CQ was the smallest carbon emitter. From the perspective of cumulative increase in IECEs, JS accounted for 20.43% of the total increase in the YREZ. The proportion exceeded 10% in AH, JX, HN and GZ. The lowest increase in IECEs was observed in ZJ and SC.

(3) The driving factors of IECEs in sub-regions were different. R&D intensity was the main promotional factor in SH. Investment intensity was the main promotional factor in HB, HN and GZ. In the other provinces, economic output was responsible for increasing IECEs.

(4) In nearly half of the provinces, the increment of IECEs changed from positive during 2008–2012 to negative during 2012–2016. This change was due to the combined less positive effects of economic output and investment intensity and the more inhibitory effect of energy intensity.

## Figures and Tables

**Table 1:** Definition of variables in Equation (2).

Variable	Definition
E_ij_	Consumption of energy j in industry sector i
E_i_	Total energy consumption of industry sector i
GDP_i_	Industrial added value of industry sector i
GDP	Gross domestic product
R	Total R&D expenditure
F	Total fixed-asset investment
P	Population
CF_ij_	CO_2_ emissions coefficient: CO_2_ emission per unit of energy j in industry sector i
ES_ij_	Energy structure: proportion of consumption of energy j in total energy consumption in industry sector i
EI_i_	Energy intensity: total energy consumption per unit of industrial added value in industry sector i
IS_i_	Industrial structure: proportion of industrial added value of industry sector i in GDP
RE	R&D efficiency: GDP per unit of total R&D expenditure
RI	R & D intensity: proportion of R&D expenditure in total fixed-asset investment
II	Investment intensity: proportion of total fixed-asset investment in GDP
EO	Economic output: GDP per capita

**Table 2:** The cumulative effect of influencing factors affecting IECEs in the sub-regions and the YREZ during 2008–2016 (unit: 10^4^ tons).

Province	Year	Δ*C_ES_*	Δ*C_EI_*	Δ*C_IS_*	Δ*C_RE_*	Δ*C_RI_*	Δ*C_II_*	Δ*C_EO_*	Δ*C_P_*	ΔC_tot_
SH	2008–2012	−256.12	−2160.35	−30.18	−3954.96	6588.11	−2633.15	914.35	2283.18	750.88
	2012–2016	−115.94	−2571.45	−112.27	−157.78	−1.23	159.01	2480.58	167.80	−151.28
JS	2008–2012	56.95	−6015.68	240.09	−12,685.21	6814.61	5870.59	9208.67	686.04	4176.06
	2012–2016	−53.35	−5822.54	−228.32	−2465.16	−1123.30	3588.46	7959.83	248.69	2104.31
ZJ	2008–2012	−121.53	−3887.65	−214.73	−9217.01	5802.03	3414.98	3611.82	817.91	205.82
	2012–2016	−289.18	−3359.28	−301.16	−2758.95	−729.94	3488.89	3560.18	261.63	−127.81
AH	2008–2012	−139.54	−2406.31	1223.84	−8738.29	5092.90	3645.38	6038.81	−252.25	4464.56
	2012–2016	−58.03	−4503.68	204.36	−3192.00	212.08	2979.92	4591.80	493.81	728.26
JX	2008–2012	−96.84	−1009.80	580.52	−2047.98	−236.38	2284.36	3380.96	170.87	3025.71
	2012–2016	−22.58	−2270.14	210.59	−3114.60	672.96	2441.64	3435.78	198.38	1552.02
HB	2008–2012	254.31	−2806.23	1659.78	−15,465.47	4807.59	10,657.88	10,169.77	265.88	9543.51
	2012–2016	−331.53	−13,516.69	162.15	−3540.57	−2560.85	6101.42	6518.44	352.04	−6815.59
HN	2008–2012	−173.80	−6092.07	1212.94	−11,513.52	4702.40	6811.12	6496.09	552.65	1995.81
	2012–2016	26.73	−2823.62	−17.52	−3033.03	−1938.16	4971.19	4737.17	413.22	2335.98
CQ	2008–2012	−25.64	−4287.45	543.27	−3488.69	1646.32	1842.38	4454.86	309.14	994.18
	2012–2016	−274.38	−3123.17	114.31	−2387.94	1274.31	1113.63	2968.76	262.65	−51.82
SC	2008–2012	−61.54	−8842.82	1980.57	−5484.38	−79.03	5563.41	9212.63	−135.24	2153.61
	2012–2016	−676.56	−7342.44	274.95	−4702.77	1156.74	3546.03	5272.69	394.17	−2077.19
GZ	2008–2012	−21.73	−1882.55	498.10	−2772.18	−3171.94	5944.12	5472.87	−758.68	3308.01
	2012–2016	−163.32	−4349.15	175.33	−1644.72	−2883.27	4527.99	4445.69	226.03	334.58
YN	2008–2012	−85.50	−3627.30	839.37	−8703.74	4848.59	3855.15	5141.57	285.65	2553.79
	2012–2016	−97.85	−4738.34	176.44	−3637.45	−815.86	4453.31	4112.29	290.90	−256.56
YREZ	2008–2012	−670.98	−43,018.20	8533.56	−84,071.42	36,815.19	47,256.23	64,102.39	4225.17	33,171.94
	2012–2016	−2055.97	−54,420.49	658.86	−30,634.98	−6736.51	37,371.49	50,083.20	3309.31	−2425.09

## Appendix A

**Table A1:** Abbreviations in the selected region.

Regions	Shanghai	Zhejiang	Jiangsu	Anhui	Jiangxi	Hubei	Hunan	Chongqing	Sichuan	Guizhou	Yunnan
Abbreviation	SH	ZJ	JS	AH	JS	HB	HN	CQ	SC	GZ	YN

## References

[1] Dong F., Li J., Zhang Y.-J., Wang Y. (2018). Drivers analysis of CO_2_ emissions from the perspective of carbon density: The case of Shandong Province, China. Int. J. Environ. Res. Public Health.

[2] Ebi K.L., Mills D.M., Smith J.B., Grambsch A. (2006). Climate change and human health impacts in the United States: An update on the results of the US national assessment. Environ. Health Perspect..

[3] Lelieveld J., Klingmüller K., Pozzer A., Burnett R.T., Haines A., Ramanathan V. (2019). Effects of fossil fuel and total anthropogenic emission removal on public health and climate. PNAS.

[4] Bi P., Hansen A. (2018). Carbon emissions and public health: An inverse association?. Lancet Planet. Health.

[5] Geng Y. (2011). Eco-indicators: Improve China’s sustainability targets. Nature.

[6] Ma X., Wang C., Dong B., Gu G., Chen R., Li Y., Zou H., Zhang W., Li Q. (2019). Carbon emissions from energy consumption in China: Its measurement and driving factors. Sci. Total Environ..

[7] Ang B.W. (2004). Decomposition analysis of policymaking in energy: Which is preferred method?. Energy Policy.

[8] Ang B.W., Liu N. (2007). Handing zero values in the logarithmic mean Divisia index decomposition approach. Energy Policy.

[9] Song M., Guo X., Wu K., Wang G. (2015). Driving effect analysis of energy-consumption carbon emissions in the Yangtze River Delta region. J. Clean. Prod..

[10] Zhang C., Su B., Zhou K., Yang S. (2019). Decomposition analysis of China’s CO_2_ emissions (2000–2016) and scenario analysis of its carbon intensity targets in 2020 and 2030. Sci. Total Environ..

[11] Zhu X.-H., Zou J.-W., Feng C. (2017). Analysis of industrial energy-related CO_2_ emissions and the reduction potential of cities in the Yangtze River Delta region. J. Clean. Prod..

[12] Xu S.-C., He Z.-X., Long R.-Y., Chen H., Han H.-M., Zhang W.-W. (2016). Comparative analysis of the regional contributions to carbon emissions in China. J. Clean. Prod..

[13] Zhang W., Jiang L., Cui Y., Xu Y., Wang C., Yu J., Streets D.G., Lin B. (2019). Effects of urbanization on airport CO_2_ emissions: A geographically weighted approach using nighttime light data in China. Resour. Conserv. Recycl..

[14] Shao S., Yang L., Gan C., Cao J., Geng Y., Guan D. (2016). Using an extended LMDI model to explore techno-economic drivers of energy-related industrial CO_2_ emissions changes: A case study for Shanghai (China). Renew. Sust. Energ. Rev..

[15] Meng Z., Wang H., Wang B. (2018). Empirical analysis of carbo emission accounting and influencing factors of energy consumption in China. Int. J. Environ. Res. Public Health.

[16] Zhao X., Zhang X., Shao S. (2016). Decoupling CO_2_ emissions and industrial growth in China over 1993–2013: The role of investment. Energy Econ..

[17] Chen C., Huang J., Chang H., Lei H. (2019). The effect of indigenous R&D activities on China’s energy intensity: A regional perspective. Sci. Total Environ..

[18] Wang J., Hu M., Rodrigues J.F.D. (2018). The evolution and driving forces of industrial aggregate energy intensity in China: An extended decomposition analysis. Appl. Energy.

[19] Zou J., Tang Z., Wu S. (2019). Divergent leading factors in energy-related CO_2_ emissions change among subregions of the Beijing-Tianjin-Hebei area from 2006 to 2016: An extended LMDI analysis. Sustainability.

[20] Peng L., Xia J., Li Z., Fang C., Deng X. (2020). Spatio-temporal dynamics of water-related disaster risk in the Yangtze River Economic Belt from 2000 to 2015. Resour. Conserv. Recycl..

[21] Ren S., Fu X., Chen X. (2012). Regional variation of energy-related industrial CO_2_ emissions mitigation in China. China Econ. Rev..

[22] IPCC Guidelines for National Greenhouse Gas Inventories. http://www.ipcc-nggip.iges.or.jp/public/2006gl/vol2.html.

[23] Li W., Zhang S., Lu C. (2019). The semi-centennial timescale dynamic assessment on carbon emission trajectory determinants for Hebei Province within the New Normal pattern shock. Sci. Total Environ..

[24] Li M., Zhang Q., Kurokawa J., Woo J.-H., He K., Lu Z., Ohara T., Song Y., Streets D.G., Carmichael G.R. (2017). MIX: A mosaic Asian anthropogenic emission inventory under the international collaboration framework of the MICS-Asia and HTAP. Atmos. Chem. Phys..

[25] Fatima T., Xia E., Cao Z., Khan D., Fan J.-L. (2019). Decomposition analysis of energy-related CO_2_ emissions in the industrial sector of China: Evidence from the LMDI approach. Environ. Sci. Pollut. Res..

[26] Guan D., Meng J., Reiner D.M., Zhang N., Shan Y., Mi Z., Shao S., Liu Z., Zhang Q., Davis S.J. (2018). Structural decline in China’s CO_2_ emissions through transitions in industry and energy systems. Nat. Geosci..

[27] Wu Y., Tam V.W.Y., Shuai C., Shen L., Zhang Y., Liao S. (2019). Decoupling China’s economic growth from carbon emissions: Empirical studies from 30 Chinese provinces (2001–2015). Sci. Total Environ..

[28] Zhang W., Stern D., Liu X., Cai W., Wang C. (2017). An analysis of the costs of energy saving and CO_2_ mitigation in rural households in China. J. Clean. Prod..

[29] Zhang W., Wang C., Zhang L., Xu Y., Cui Y., Lu Z., Streets D.G. (2018). Evaluation of the performance of distributed and centralized biomass technologies in rural China. Renew. Energy.

[30] Zhang W., Lu Z., Xu Y., Wang C., Gu Y., Xu H., Streets D.G. (2018). Black carbon emissions from biomass and coal in rural China. Atmos. Environ..

[31] Gu Y., Zhang W., Yang Y., Wang C., Streets D.G., Yim S.H.L. (2020). Assessing outdoor air quality and public health impact attributable to residential black carbon emissions in rural China. Resour. Conserv. Recycl..

